# Biobased Terpene Derivatives: Stiff and Biocompatible Compounds to Tune Biodegradability and Properties of Poly(butylene succinate)

**DOI:** 10.3390/polym14010161

**Published:** 2021-12-31

**Authors:** Reza Zeinali, Luis J. del Valle, Lourdes Franco, Ibraheem Yousef, Jeroen Rintjema, Carlos Alemán, Fernando Bravo, Arjan W. Kleij, Jordi Puiggalí

**Affiliations:** 1 Departament d’Enginyeria Química, EEBE, Universitat Politècnica de Catalunya, 08019 Barcelona, Spain; reza.zeinali@upc.edu (R.Z.); lourdes.franco@upc.edu (L.F.); carlos.aleman@upc.edu (C.A.); 2 Center for Research in Nano-Engineering, CrNE, Universitat Politècnica de Catalunya, C. Eduard Maristany, 08019 Barcelona, Spain; 3 ALBA Synchrotron Light Source, Carrer de la Llum, 2-26, Cerdanyola del Vallès, 08290 Barcelona, Spain; iyousef@cells.es; 4 ICREA—Catalan Institution for Research and Advanced Studies, 08010 Barcelona, Spain; jrintjema@iciq.es; 5 Institute of Chemical Research of Catalonia (ICIQ), Barcelona Institute of Science and Technology, 43007 Tarragona, Spain; fbravo@iciq.es (F.B.); akleij@iciq.es (A.W.K.); 6 Institute for Bioengineering of Catalonia, Baldiri Reixac 10-12, 08028 Barcelona, Spain

**Keywords:** biobased materials, terpene derivatives, biodegradability, thermal properties, spherulites, scaffolds, thermally-induced phase separation

## Abstract

Different copolymers incorporating terpene oxide units (e.g., limonene oxide) have been evaluated considering thermal properties, degradability, and biocompatibility. Thus, polycarbonates and polyesters derived from aromatic, monocyclic and bicyclic anhydrides have been considered. Furthermore, ring substitution with myrcene terpene has been evaluated. All polymers were amorphous when evaluated directly from synthesis. However, spherulites could be observed after the slow evaporation of diluted chloroform solutions of polylimonene carbonate, with all isopropene units possessing an R configuration. This feature was surprising considering the reported information that suggested only the racemic polymer was able to crystallize. All polymers were thermally stable and showed a dependence of the maximum degradation rate temperature (from 242 °C to 342 °C) with the type of terpene oxide. The graduation of glass transition temperatures (from 44 °C to 172 °C) was also observed, being higher than those corresponding to the unsubstituted polymers. The chain stiffness of the studied polymers hindered both hydrolytic and enzymatic degradation while a higher rate was detected when an oxidative medium was assayed (e.g., weight losses around 12% after 21 days of exposure). All samples were biocompatible according to the adhesion and proliferation tests performed with fibroblast cells. Hydrophobic and mechanically consistent films (i.e., contact angles between 90° and 110°) were obtained after the evaporation of chloroform from the solutions, having different ratios of the studied biobased polyterpenes and poly(butylene succinate) (PBS). The blend films were comparable in tensile modulus and tensile strength with the pure PBS (e.g., values of 330 MPa and 7 MPa were determined for samples incorporating 30 wt.% of poly(PA-LO), the copolyester derived from limonene oxide and phthalic anhydride. Blends were degradable, biocompatible and appropriate to produce oriented-pore and random-pore scaffolds via a thermally-induced phase separation (TIPS) method and using 1,4-dioxane as solvent. The best results were attained with the blend composed of 70 wt.% PBS and 30 wt.% poly(PA-LO). In summary, the studied biobased terpene derivatives showed promising properties to be used in a blended form for biomedical applications such as scaffolds for tissue engineering.

## 1. Introduction

The use of partially or fully biobased materials is receiving increasing attention to attain a gradual/complete substitution of petroleum-based resources [1,2]. Microorganisms, plants or algae can provide different biobased polymers (e.g., lignin, cellulose, sugar, starch and poly(hydroxyalkanoate)s (PHAs)); however, the majority of them are ex vivo produced from biological-based monomers (e.g., natural oils, fatty acids, glycols) [3]. The composition of PHAs supports various combinations and, based on the biotechnological production strategy (microbial growth substrate and metabolic pathway used), can be obtained as homopolyesters or heteropolyesters. This high variability justifies the wide range of potential applications of PHAs with a biodegradable and biocompatible nature. In fact, the high potential of PHAs as an effective alternative to replace synthetic plastics is a hot topic [4].

The chemistry of functional terpenes provides an interesting option to obtain new biopolymers (e.g., polyterpenes [5] and polycarbonates [6,7]). Ring-opening copolymerization can also be employed to prepare related polyesters from aliphatic cyclic esters/anhydrides and epoxides [8,9]. Great interest is focused on the use of limonene, a commercial monoterpene that can easily be isolated from citrus fruits [10]. Epoxidation of the double bond belonging to the cycle gives rise to reactive limonene oxide (LO), which can be used as a platform molecule to obtain polycarbonates, polyesters, polyurethanes and even polyamides [11]. Terpene oxide monomers (e.g., limonene oxide (LO) and menthene oxide (MO)) can be copolymerized in a ring-opening copolymerization (ROCOP) process with cycloaliphatic anhydrides (e.g., *cis*-4-cyclohexene-1,2-dicarboxylic anhydride (MA)) and aromatic anhydrides (e.g., phthalic anhydride (PA)). The incorporation of rigid cycles appears a promising strategy for the development of high-*T_g_* biobased polymers [12,13] and may even be interesting in other sectors where polycarbonates are highly promising [14].

Polybutylene succinate (PBS) is an aliphatic polyester with excellent mechanical and thermal properties [15,16]. In fact, PBS is considered an interesting substitution for polyolefins in some applications due to both its good performance and its easy processing characteristics. PBS is produced by the condensation of succinic acid and 1,4-butanediol monomers, which can either be derived from fossil fuels or bacterial fermentation. Several companies around the world are precisely developing technologies to produce biobased polymers [17,18]. PBS has been employed as mulch films, flushable hygiene products, foaming, packaging and even biomedical applications [19,20]. The polymer has also been blended with different polymers (e.g., polylactide and starch) to improve some properties (e.g., softness and barrier properties) [21,22]. Thus, PBS blended with furfural as a natural component showed a clear improvement in mechanical properties while the biobased content was increased [23].

Biocompatible and biodegradable scaffolds are continuously being developed to satisfy the different requirements that are needed in the field of tissue engineering. In this sense, efforts are involved to expand the range of suitable polymers, including blends and composites, and the applied methodology. In recent years, thermally-induced phase separation (TIPS) has widely been used to fabricate three dimensional (3D) porous scaffolds. The main advantages are their low production cost, capacity to control the final architecture and its experimental simplicity [24]. The preparation of PBS scaffolds from TIPS has recently been studied in detail, demonstrating the ability to produce both random and oriented pore structures using 1,4-dioxane as a solvent [25].

**Figure 1:**
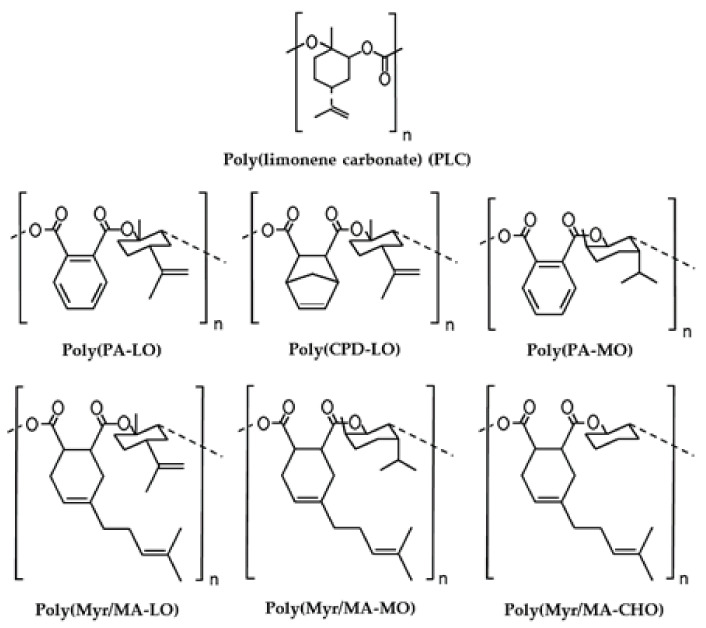
Chemical structure and abbreviations of the different studied terpene derivatives.

**Figure 2:**
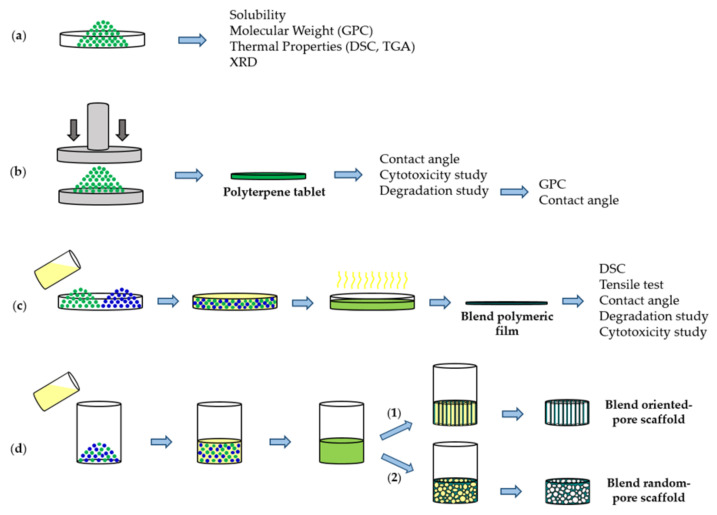
Graphical scheme of study design. Green biobased terpene derivative polymer powder from synthesis (**a**). Polymeric tablet obtained by cold-pressing of polymer powder (**b**). Blend (poly(PA-LO)/PBS) polymeric film prepared by solvent casting using chloroform solvent (**c**). Oriented-pore and random-pore blend (poly(PA-LO)/PBS) scaffold fabricated by TIPS technique using 1,4 dioxane solvent and uniaxial (1) and multidirectional (2) thermal gradient, followed by solvent removal via freeze-drying (**d**).

The present work is focused on the preparation of new PBS blends with biobased terpene derived polymers and explore the application of a selected composition to obtain scaffolds by the TIPS technique. To this end, polylimonene carbonate and different copolyesters with low molecular weight will be considered in order to facilitate subsequent absorption by the organism (Figure 1). All samples were prepared from previously reported procedures [6,26,27,28,29,30,31]. Polymers were based on two terpene oxide monomers (i.e., LO and MO), different highly rigid anhydrides based on the aromatic phthalic unit and monocyclic or bicyclic aliphatic units with internal double bonds. Furthermore, in some cases, the terpene myrcene was incorporated as a lateral group of the corresponding cycle to expand the range of properties. It is expected that the selected terpene biobased components increase the rigidity of the final scaffold, reduce the crystallinity due to their amorphous character, deaccelerate degradation due to their reduced chain mobility and can be absorbed after the implantation period. A graphical scheme summarizing the different steps involved in the present work is summarized in Figure 2.

## 2. Experimental

### 2.1. Materials

The polybutylene succinate (PBS) used in this study was a commercial product (Bionolle^®^ 1001 MD) supplied by Showa Denko K.K. (München, Germany). This polymer has a melt flow index of 1.6 g/10 min, measured at 190 °C under a load of 2.16 Kg according to ASTM-D1238. Copolymers with terpene units were synthesized in the Institute of Chemical Research of Catalonia (ICIQ).

1,4-dioxane and chloroform with the purity of 99.5% were purchased from Acros Organics(Fisher Scientific S.L., Madrid, Spain) and used as received. Lipase enzyme (L3126) from porcine pancreas, esterase (E-3019) from porcine liver crude and boric acid were purchased from Sigma-Aldrich (Saint Louis, MO, USA).

### 2.2. Measurements

Molecular weight was estimated by size exclusion chromatography (GPC) using a liquid chromatograph (Shimadzu, model LC-8A, Kyoto, Japan) equipped with an Empower computer program (Waters). A PL HFIP gel column (Polymer Lab) and a refractive index detector (Shimadzu RID-10A, Kyoto, Japan) were employed. The polymer was dissolved and eluted in 1,1,1,3,3,3-hexafluoroisopropanol (HFIP) containing CF_3_COONa (0.05 M) at a flow rate of 0.5 mL/min (injected volume 100 μL, sample concentration 2.0 mg/mL). The number and weight average molecular weights were calculated using poly(methyl methacrylate) standards.

Calorimetric data were obtained by differential scanning calorimetry (DSC) with the TA Instruments (New Castle, DE, US) Q100 series equipped with a refrigerated cooling system (RCS) operating at temperatures from −80 °C to 400 °C. Calibration was performed with Indium. Experiments were conducted under a flow of dry nitrogen with a sample weight of approximately 5 mg. Calorimetric data were obtained from runs performed at a heating rate of 10 °C/min.

The thermal stability of the terpene derivatives was studied by thermogravimetric analysis (TGA) at a heating rate of 10 °C/min (sample weight ca. 5 mg) with a Q50 thermogravimetric analyzer from TA Instruments and under a flow of dry nitrogen. Test temperatures ranged from 30 to 600 °C.

The sessile drop method was used to determine the contact angle (CA) between polymer specimens (rectangular tablets and solvent casting films with dimensions of 3 cm × 1 cm) and milliQ water drops (0.5 µL). Measurements were taken with OCA 15EC 146 equipment (DataPhysics Instruments GmbH, Filderstadt, Germany), and SCA20 software was employed to determine the CA from an average of more than 40 measures.

A Zwick Z2.5/TN1S (Zwick Roell Group, Ulm, Germany) testing machine was employed to perform stress–strain tests. The crosshead rate and load cell capacity were 150 mm/min and 100 N, respectively. Parameters were determined using the TestXpert software of Zwick and were averaged from six independent measurements for each sample. Tested specimens were cut according to rectangular shapes (3 cm × 0.4 cm) from solvent casting films (thickness of 0.40 mm).

The porous structure of the polymer matrices was evaluated using a scanning electron microscope (SEM) (Focus IonBeam Zeiss Neon 40 instrument, Carl Zeiss, Oberkochen, Germany) at 5 kV and controlled by Smart Tiff software (Carl Zeiss SMT Ltd., Oberkochen, Germany). To obtain the original cross-sectional parts, the samples were fractured in liquid nitrogen after being soaked for several minutes. The top surfaces and cross-sections of the samples were coated with carbon for 700 s using a sputter coater (Mitec K950 Sputter Coater, Quorum Technologies Ltd., Ashford, UK) before microscopy was performed.

X-ray powder diffraction patterns were obtained with a PANalytical X’Pert diffractometer with CuΚ_α_ radiation (λ = 0.1542 nm) and a Silicon monocrystalline sample holder. Deconvolution of WAXD peaks was performed using the PeakFit 4.0 program.

Microspectroscopic measurements were performed at the infrared beamline MIRAS of the ALBA synchrotron. Spherulites were grown by slow evaporation onto transparent CaF_2_ windows (13 mm diameter). Observations were performed in a transmission mode using a Hyperion 3000 microscope coupled with a Vertex 70 spectrometer (Bruker Corp., Billerica, MA, US). The spectral resolution was 4 cm^−1^ with 256 co-added scans per spectrum. Light was also polarized at 0° or 90° by a ZnSe holographic wire grid polarizer (Acal BFi, Zaventem, Belgium).

### 2.3. Degradation Studies

The degradation study was performed using different media at 37 °C, including hydrolytic media with pH 3, 7 or 10, lipase or esterase enzymatic media and oxidative media containing 30% H_2_O_2_. The degradation in basic media with pH 10 was also performed at 70 °C.

The samples for degradation studies were prepared in the form of polymeric tablets using a pressing machine. After preparation, the samples were exactly weighed. For statistical calculations, at least three samples were prepared for each degradation condition/period.

The universal Britton–Robinson buffer was used to obtain different pH media following the procedure described in the literature [32]. A 10 mM borate buffer was prepared using boric acid and deionized water by adjusting the solution to pH 8.0 with 1 M NaOH. In the case of the universal buffer media with pH 10, the samples were also placed in a 70 °C oven.

For enzymatic media, lipase was dissolved in phosphate-buffered saline at the concentration of 1% (*w*/*v*), while esterase was dissolved in the borate buffer at the concentration of 50 units/mL. In total, 2 mL from each degradation media was added to the pre-weighed polymeric tablets, and the samples were placed in the incubator at 37 °C.

During the degradation study, the samples were sealed to prevent evaporation and the media was replaced to ensure freshness three times a week (i.e., every 2–3 days). Every 7 days, a group of samples were removed from the incubator. These samples were washed with distilled water, then the surface was well dried with a paper and finally, the sample was placed in the 37 °C desiccator until a constant weight was reached (usually 48 h). After drying, the samples were weighed on two consecutive days. If the weight difference was less than 5%, it was registered as the final weight of the sample, corresponding to its degradation period.

The weight loss ($w_{l}$) of the specimens was determined through Equation (1), where $w_{d}$ is the sample weight after degradation and $w_{0}$ is the initial sample weight, i.e., before exposure to the degradation medium:

(1) $$w_{l}\left(\%\right)=\left(\frac{w_{0}-w_{d}}{w_{0}}\right)100$$

### 2.4. Cell Adhesion and Proliferation Assays

Studies were performed with fibroblast hFF cells (human foreskin fibroblasts, HFF-1 ATCC) and fibroblast MG-63 cells (derived from human osteosarcoma, CRL-1427, ATCC). In all cases, cells were cultured in Dulbecco’s Modified Eagle Medium (DMEM) with 4500 mg/L of glucose, 110 mg/L of sodium pyruvate, 2 mM of L-glutamine supplemented with 10% fetal bovine serum (FBS), 50 U/mL penicillin, 50 mg/mL streptomycin and L-glutamine 2 mM at 37 °C in a 10% humidified atmosphere of 5% CO_2_ and 95% air. Culture media were changed every two days. For sub-cultures, cell monolayers were rinsed with phosphate-buffered saline and detached by incubating them with 0.25% trypsin/EDTA for 2–5 min at 37 °C. The incubation was stopped by suspending in 5 mL of fresh medium. The cell concentration was determined by counting with Neubauer camera and using 4% trypan blue as dye vital.

Square pieces of films (1 cm × 1 cm) with a thickness of 0.1 cm were prepared by solvent-casting and fixed in each well of a 24-well culture plate with a small drop of silicone (Silbione^®^ MED ADH 4300 RTV, Bluestar Silicones France SAS, Lyon, France).

The plate was sterilized by UV radiation for 15 min using a laminar flux cabinet. Cell adhesion assays were carried out using aliquots of 50–100 μL containing 5 × 10^4^ cells. These aliquots were seeded onto the polymeric pieces placed in each well, and incubated for 24 h. Cell proliferation assays were performed similarly, but the aliquots contained only 2 × 10^4^ cells (i.e., a lower cell concentration than used for adhesion), and the incubation time increased to 96 h. Quantitative evaluation of adhesion and proliferation was performed according to the MTT method [33,34], which is based on a simple modification of the ISO10993-5:2009 standard test. Viability results were averaged from four replicates.

### 2.5. Scaffold Preparation

A thermally-induced phase separation (TIPS) fabrication process with a one-step cooling protocol was applied for the preparation of PBS/poly(PA-LO) scaffolds. The selected polymer mixture (70 wt.% of PBS) was dissolved in 1,4-dioxane at a concentration of 2.5% (*w/v*) and a temperature of 70 °C. The initially homogeneous solution underwent phase separation by decreasing temperature, which led to porous matrices after solvent removal. Specifically, the polymer solutions were poured into cylinder-shaped glass vials with a diameter of 14 mm and height of 40 mm and then carefully sealed to avoid evaporation. Samples were immediately incorporated into the corresponding cooling devices, that is, two freezers with pre-set temperatures of −20 °C and −74 °C and also a liquid nitrogen bath (−196 °C). The cooling process was performed under different thermal gradients, that is, from a relatively high temperature at which the solution is still clear and homogenous to the temperature of the selected baths. To apply multidirectional temperature gradient (i.e., a randomly-oriented cooling system), the glass molds were directly immersed in −20 °C or −74 °C baths. Whereas to apply a uniaxial temperature gradient (i.e., a uniaxially-oriented cooling system), the glass vials were inserted into a central hole of a polystyrene mold in order to insulate the perimeter walls of the vials and prevent the heat transfer through the side walls. In this case, only the bottom surface of the vials touched the −74 °C and −196 °C cooling baths. After remaining for 24 h in the −20/−74 °C or 15 min in −196 °C cooling baths, the samples were freeze-dried for a 120 h period using a Virtis^®^ 6201-3120 lyophilizer (Gardiner, NY, USA). The obtained porous matrices were then dried in a vacuum desiccator at room temperature to reach a constant weight.

### 2.6. Statistical Analysis

Experimental values were averaged and graphically represented together with their respective standard deviations. Statistical analysis was performed by one-way ANOVA test to compare the means of all groups, and then Tukey’s test was applied to determine a statistically significant difference between the two groups. The test confidence level was set at 95% (*p* < 0.05).

## 3. Results and Discussion

### 3.1. Solubility, Molecular Weight and Hydrophobicity of the Biobased Terpene Derivatives

All synthesized biobased terpene derivatives were highly soluble in typical organic solvents (e.g., CHCl_3_, 1,4-dioxane, THF, DMSO) and strong fluorinated acids such as trifluoroacetic acid. The main differences concerned formic acid and alcohols such as ethanol and even methanol (Table 1).

Note that compounds having rings with lateral myrcene groups were the most soluble in alcohols, probably as a consequence of the difficult packing caused by these big groups. Note that all compounds were highly soluble in 1,4-dioxane, a solvent that was then selected for the preparation of scaffolds by TIPS.

Table 2 show the GPC data of polymers directly obtained from synthesis. In agreement with the synthesis procedure, the characteristic molecular weights were low (i.e., average molecular weights between 7600 and 9700 g/mol) and had a uniform distribution since polydispersity indices varied between 2.1 and 2.5. GPC curves for representative samples with the highest and lowest molecular weight are shown in Figure S1. Note that a significant mistake in the molecular weight estimation is expected due to the use of the more flexible poly(methyl methacrylate) polymer as a calibrant.

Low molecular weights meant that only very brittle or powdered films were obtained from the different samples obtained by solvent casting and specifically using chloroform as solvent. As will be explained, consistent films were only obtained after blending with PBS (Figure S2).

All samples could be considered slightly hydrophobic since contact angles were equal to or higher than 90°. No significant variation was detected, with the greatest and lowest values being 100° and 90° (Figure S3), respectively.

Note that contributions to hydrophilicity derived from terminal groups were practically identical for all samples due to their similar molecular weights. It should also be pointed out that the sample preparation method was identical, and the hydrophobic roughness contribution should also be similar. Therefore, the different modifications of the chemical repeat unit did not render an appreciable change on the surface hydrophobicity. Note that maximum and minimum contact angles correspond to highly similar samples derived from phthalic anhydride and terpene oxide, while intermediate values were determined for the rest of the samples.

### 3.2. Thermal Properties of Studied Biobased Polymers

**Figure 3:**
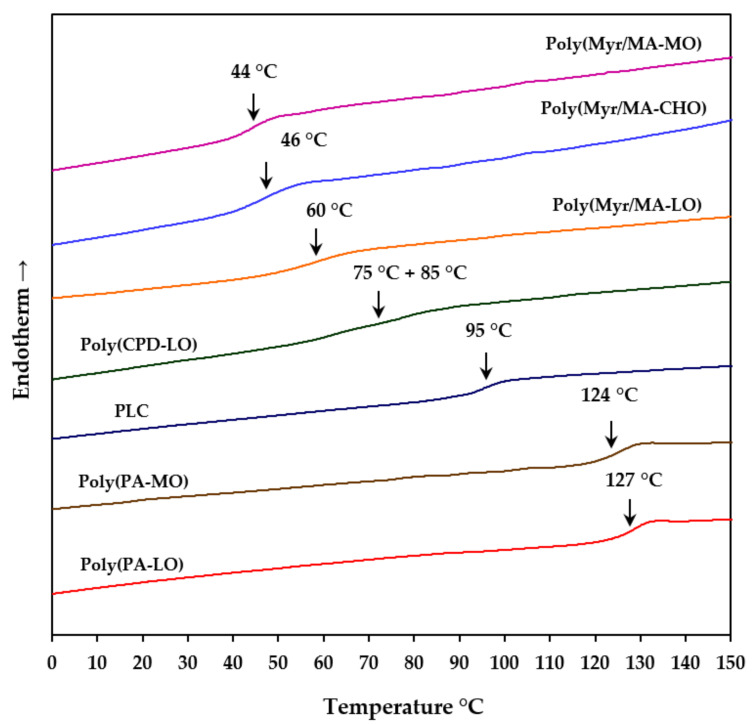
DSC heating runs of the synthesized terpene derivatives.

DSC heating runs of the studied biobased terpene derivatives were indicative of fully amorphous samples. Thus, only a well-defined glass transition was detected, and no evidence of a minor melting peak was observed even for PLC, which theoretically is the more crystalline sample. Figure 3 show that the *T*_g_ varies in a wide temperature range (i.e., from 44 °C to 127 °C), corresponding the lower values to those polymers having myrcene lateral groups.

These groups are responsible for certain mobility and will be discarded for further applications according to the proposed goals. The higher *T*_g_ values (i.e., 127 °C and 124 °C) were attained with the copolymers derived from phthalic anhydride, having a more rigid structure that incorporates LO units. It should also be indicated that PLC showed an interesting high value (95 °C), whereas the copolymer with the bicyclic unit seems to have two close *T*_g_ at 75 °C and 85 °C.

**Figure 4:**
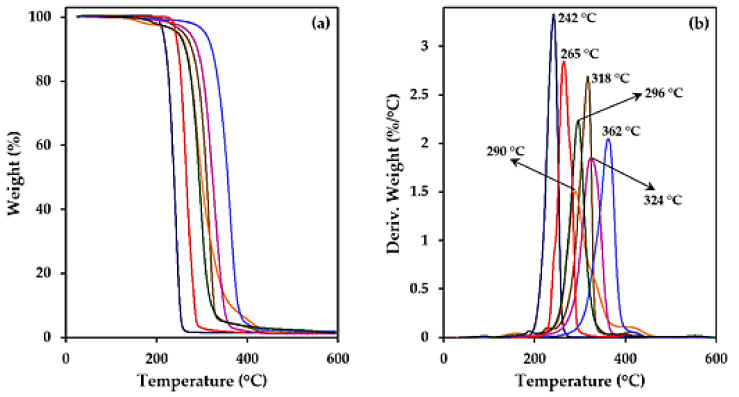
TGA (**a**) and DTGA (**b**) curves for the different studied terpene derivatives. Peak temperature for DTGA curves increased in the order: PLC, poly(PA-LO), poly(Myr/MA-LO), poly(CPD-LO), poly(PA-MO), poly(Myr/MA-MO), poly(Myr/MA-CHO). The same colour code has been applied for TGA curves.

All the studied polyterpene derivatives had high thermal stability, as demonstrated by the corresponding TGA and DTGA curves (Figure 4). PLC was the less stable sample with an onset of degradation lower than 200 °C and a DTGA peak at 242 °C.

The onset temperature was higher than 200 °C for the different studied copolyesters, being the decomposition peak mainly related to the nature of the oxide unit (i.e., LO, MO or cyclohexene oxide (CHO)). Thus, the peak temperature decreased in the order CHO > MO > LO (i.e., 362 °C, 324–318 °C and 296–265 °C, respectively).

With respect to the nature of the dicarboxylic unit (i.e., aromatic, aliphatic bicycle/monocycle, myrcene substituted cycles), a minor effect was detected. Note, for example, that peak temperatures of 290 °C and 296 °C were determined for polymers with highly different repeat units but coming from the same terpene oxide (i.e., poly(Myr/MA-LO) and poly(CPD-LO)). Degradation curves suggest a single step thermal decomposition process with the exception of the poly(Myr/MA-LO) sample characterized by at least three different decomposition steps. PLC, the polymer with the simplest repeat unit, had the narrowest DTGA curve. In this case, degradation could be described by a single mechanism. Degradation was completed for all samples at 450 °C with a char yield lower than 5%.

### 3.3. Remarks on the Crystallization of Polylimonene Carbonate

Copolymers of limonene oxide and carbon dioxide have a chiral carbon atom (i.e., that linked to the isopropenyl substituent). Thus, two enantiomeric forms (poly(1S,2S,4R-limonene carbonate) and poly(1R,2R,4S-limonene carbonate) can be prepared using, for example, an Al-(aminotriphenolate) complex as a catalyst [6,30,31]. It has been reported that both enantiomers are unable to crystallize despite having regular constitution and conformation. Interestingly, the racemic mixture gave rise to a stereocomplex and an interdigitated structure composed of sheets with an alternating disposition of chains with an opposite chirality [7]. A chain periodicity of 1.131 nm and a 2/1 helical conformation with nearly all-trans bonds in the backbone was postulated. Nevertheless, some conformational disorder was even observed for this favorable crystalline arrangement. Specifically, problems were related to the easy twisting of cyclohexane rings, the low conformational barrier of bonds connecting the isopropenyl groups to the ring and the statistical occupancy of up and down chains in the lattice [35]. Crystallization of the enantio-pure form was problematic due to the weak interactions between molecular chains having an isochiral packing that led to low crystallization kinetics. In fact, it was reported that amorphous samples were always obtained despite exploring different precipitation and solution casting procedures and using polymers of very low molecular weight (e.g., 9000 g/mol) [29].

**Figure 5:**
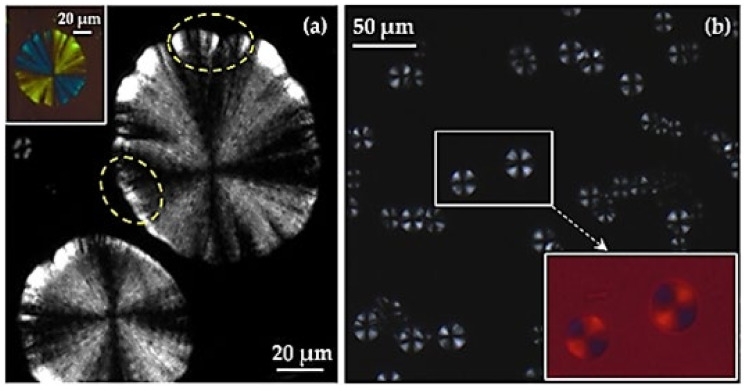
Spherulitic morphologies of PLC attained during slow evaporation of diluted chloroform solutions. Borders and sizes point out the crystallization of fractions with low (**a**) and medium (**b**) molecular weights.

In the present work, we can report the possibility to obtain typical spherulitic morphologies from a non-regular chain configuration (i.e., the isopropene units have an R configuration, but the carbons bearing the carbonate links can have a cis/trans relation with reference to isopropene) by the slow evaporation of diluted chloroform solutions (i.e., 0.1 g/mL). This slow rate was achieved when drops of the solution were placed between a slip and a coverslip and led to evaporation at room temperature. The predominant morphology (Figure 5a) corresponded to ringed spherulites having diameters and interring spacings close to 80 µm and 5 µm, respectively. This negative birefringence is usually observed in polymers, including polyesters and polycarbonates. The maximum refractive index of the corresponding crystalline assemblies is associated with the chain molecular axis, which corresponds to the tangential direction of typical spherulites constituted by edge-on lamellae that radially grow from the crystalline nucleus.

A ringed texture is usually associated with the twisting of the constitutive edge-on lamellae [36], but other interpretations have also been postulated (e.g., development of interlamellar screw dislocation [37] or the presence of different polymorphic structures [38]). However, the crystallization derived as a result of slow evaporation from dissolution and the low sample molecular weight could also be related to a rhythmic crystal growth derived from the presence of depletion zones in the growth front [39,40]. It has been postulated that the final spherulitic morphology is the consequence of a balance between the diffusion rate (*v_d_*) of polymer chains and the crystallization rate (*v_c_*) [40,41,42]. Ridge and valley topographic textures can therefore be observed depending on the special crystallization conditions [40].

It is highly interesting that all spherulites also have an umbrella-like geometry, which appears characteristic of the crystallization of low molecular weight polymers [43]. This umbrella-like or rose-like morphology has been discussed in detail by Okui et al. [44] for melt crystallization performed at high temperatures. The rosaceous growth was related to the instability of the crystal growth front in thin-film samples (a common feature for a crystallization performed from a diluted solution) [45]. Crystallization could begin with the growth of edge-on lamellae, but before attaining the typical spherical envelope, a growth with flat-on lamellae is favoured. This can give rise to hedrites and rose-like crystals that contrast with the typical morphologies expected for the edge-on lamellae [46,47]. Note also in Figure 4a the high birefringence of the spherulite limits, appearing even brilliant in some parts of the theoretically non-birefringent arms of the Maltese cross (see dashed ellipsoid).

A second but minor type of spherulites can also be detected (Figure 5b) during the evaporation process. In this case, the diameter is reduced to 20 µm, but both birefringence and ringed texture can be detected. The main difference corresponds to the rounded morphology, which, together with the indicated small size of spherulites, point out to the crystallization of a fraction of molecules with a bigger molecular weight. Nevertheless, this hypothesis is not supported by GPC data. In any case, the perfect spherical envelope and the clear rings (spacing close to 2 µm) suggest a twisting of edge-on lamellae.

**Figure 6:**
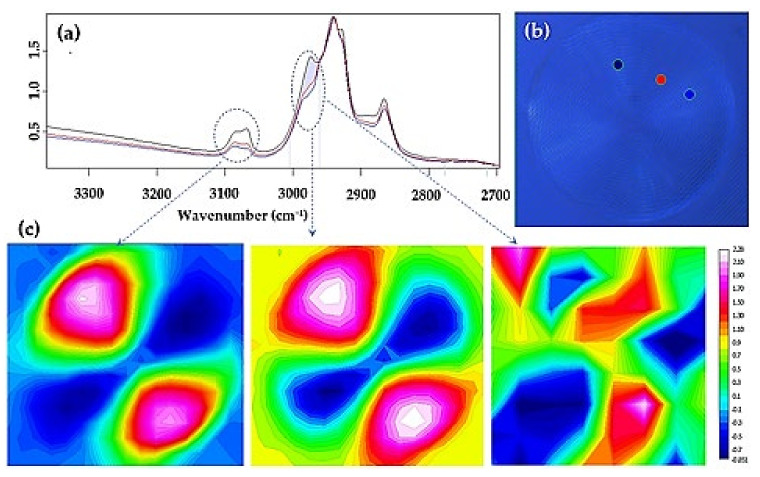
(**a**) FTIR spectra showing the C–H stretching region for PLC and taken from three representative microdomains of the spherulite. (**b**) Micrograph showing a representative banded spherulite and the specific microdomains where FTIR spectra were recorded. Colors are in agreement with those of the FTIR spectra. (**c**) Chemical images obtained from the FTIR bands pointed out by the circle and the ellipsoid. Left and middle images correspond to the bigger spherulites, while the right image is derived from the smaller spherulites.

Chemical images of the spherulites were obtained from the integration of the different infrared peaks (Figure 6a). In general, spherulitic sectorization was not observed in the chemical images derived from the most intense bands associated with the cyclohexane rings, as, for example, the asymmetric and symmetric stretching of the CH_2_ group. In this case, the intensity of the peaks was practically constant. However, a detailed analysis was successful, and clear sectorization was observed by considering some minor intensity peaks as the high wavenumber shoulder at 2980 cm^−1^ and the bands around 3080 cm^−1^ (Figure 6a,b). The former band is assigned to CH_3_ asymmetric stretch vibration, and the latter could be ascribed to the C–H stretch.

These results point out a specific orientation of these units inside the lamellar crystals. Maltese crosses with two sectors with higher intensity (red) and two sectors with the lower intensity (blue) are derived as a consequence of the distinct lamellar orientation in the spherulite (i.e., +45° and −45° with respect to the polarizer). Sectorization was clear in the imaging of the large spherulites associated with the low molecular weight fraction (left and middle images of Figure 6c). Puzzling results (see the right image of Figure 6c) were achieved when the smaller spherulites were analyzed. Nevertheless, the successful imaging results are interesting since they reveal the role of attaining a proper and regular packing of isopropenyl groups for developing PLC crystals.

Figure S4 show the X-ray diffraction profiles obtained with the different synthesized samples. In all cases, even for the more regular PLC copolymer, broad peaks associated with an amorphous phase were observed. These usually low intense and broad humps are indicative of characteristic distances related to a low-range order. All the studied samples showed three peaks (at approximately 0.754 nm, 0.505 nm and 0.211 nm) with different relative intensities (note, for example, the different intensity ratio between the two first halos of poly(CPD-LO) and poly(PA-MO) samples. More crystalline patterns could only be observed in the case of PLC when the sample was obtained by slow evaporation from the chloroform solution.

**Figure 7:**
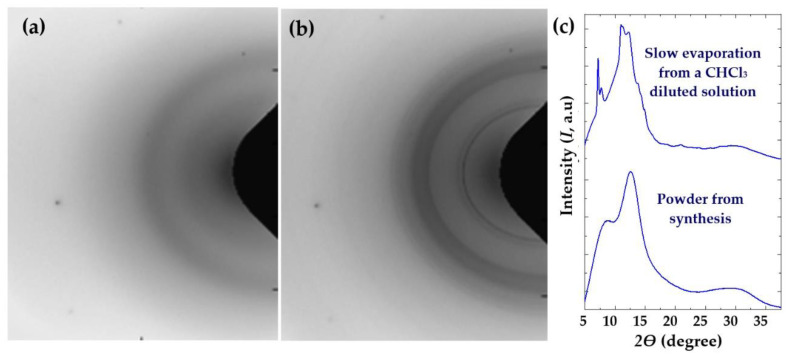
Bidimensional X-ray diffraction patterns of PLC as obtained from synthesis (**a**) and after slow evaporation of a chloroform solution (**b**). The corresponding linear X-ray profiles are given in (**c**).

Figure 7 clearly show the splitting of the indicated halos into different Bragg reflections. Those more intense correspond to spacings of 0.880 nm, 0.852 nm, 0.572 nm and 0.520 nm. Note that these values are different to those reported for the stereocomplex sample, which corresponds to the spacings of 0.915−0.907 nm and 0.483-0.477 nm [7], and logically a different packing arrangement should be derived. A monoclinic or triclinic unit cell seems necessary if the molecular chain conformation is retained.

### 3.4. Degradability of the Selected Terpene Derivatives

**Figure 8:**
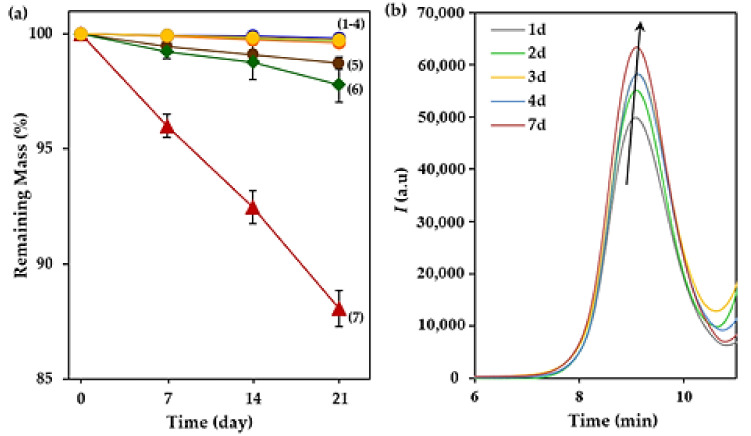
(**a**) Degradation of poly(PA-LO) as a function of time at different conditions: Hydrolytic degradation at pHs 3, 7 and 10 (curves 1–3), lipase enzymatic degradation (curve 4) and esterase enzymatic degradation (curve 5) at 37 °C, degradation in the basic pH 10 medium at 70 °C (curve 6) and degradation in oxidative media of H_2_O_2_ at 37 °C (curve 7). Weight losses were close to 0.5%, 1.5%, 2.7% and 12% for curves 1–4, curve 5, curve 6 and curve 7, respectively. (**b**) GPC curves taken for poly(PA-LO) during exposure to the esterase enzymatic medium.

All the studied polycarbonates and copolyesters were stable after exposure for 21 days to both hydrolytic and enzymatic media according to weight loss measurements. Figure 8a show the plot of the time evolution of the remaining weight for poly(PA-LO) as a representative sample. The observed changes were also minimum for the other studied samples. Hydrolytic degradation was similar for acidic, neutral and basic pHs at 37 °C, and only the increase of temperature to 70 °C caused small evidence of degradation under the more aggressive basic conditions. Lipase and esterase enzymatic media did not cause any significant degradation, although esterase was slightly more effective. The addition of H_2_O_2_ gave rise to an oxidative medium that was relatively effective since at least a weight loss higher than 10% was observed after 21 days of exposure. Nevertheless, GPC curves of exposed samples showed a slight shift to higher retention times as evidence of certain degradation. Note the evolution of GPC curves (Figure 8b) for the representative poly(PA-LO) sample during exposure to the esterase enzymatic medium. Thus, initial *M_n_* and *M_w_* values of 4000 and 9200 g/mol changed to 3060 and 7340 g/mol, respectively, after only seven days of exposure. Molecular weights of 2300 and 6100 g/mol were measured after 21 days, a feature that indicates an increase of the polydispersity index from 2.3 to 2.7. Therefore, it can be concluded that weight loss measurements did not report clear evidence of degradation due to the small solubility of fragments in the degradation medium. The low weight loss detected and the more significant change in the molecular weight seem to point out a random scission of carbonate or ester groups instead of a stepwise process.

The performed studies demonstrated that although samples could be considered degradable, the rate was slow, and consequently, the integrity of the polymer was kept during at least three weeks of exposure to both hydrolytic and enzymatic media.

**Figure 9:**
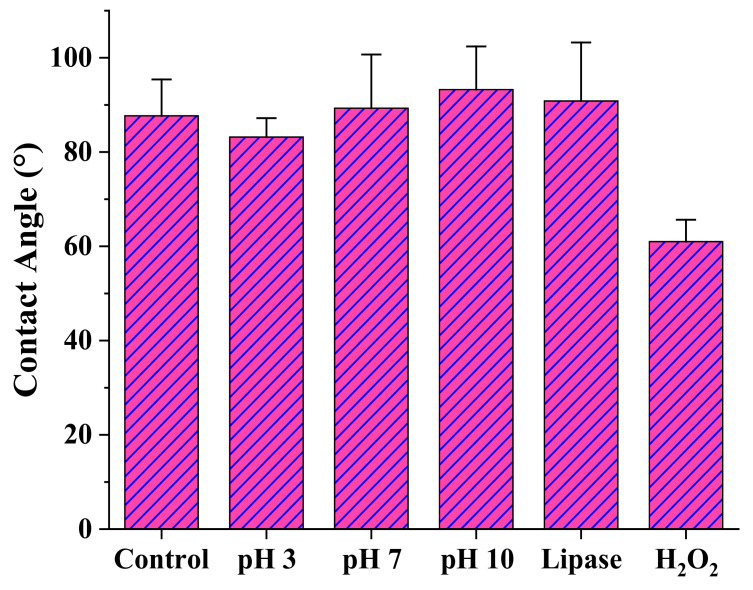
Contact angle measurements over the surface of poly(PA-LO) tablets after exposure to the indicated media for 21 days.

Surface degradation could also be detected through contact angle measurements, although interpretation is again difficult as a consequence of two opposite effects: First, the presence of more hydrophilic groups (e.g., carboxylic groups derived from the hydrolysis of ester groups) and second, the increase of surface roughness. Thus, a continuous increase in the contact angle was observed when the pH of the medium was increased, a feature that we attributed to an enhanced degradation that affected the surface roughness. By contrast, the angle significantly decreased to 60° after exposure to the oxidative medium due to the significant increase of terminal groups (Figure 9).

### 3.5. Biocompatibility of the Studied Biobased Polymers

**Figure 10:**
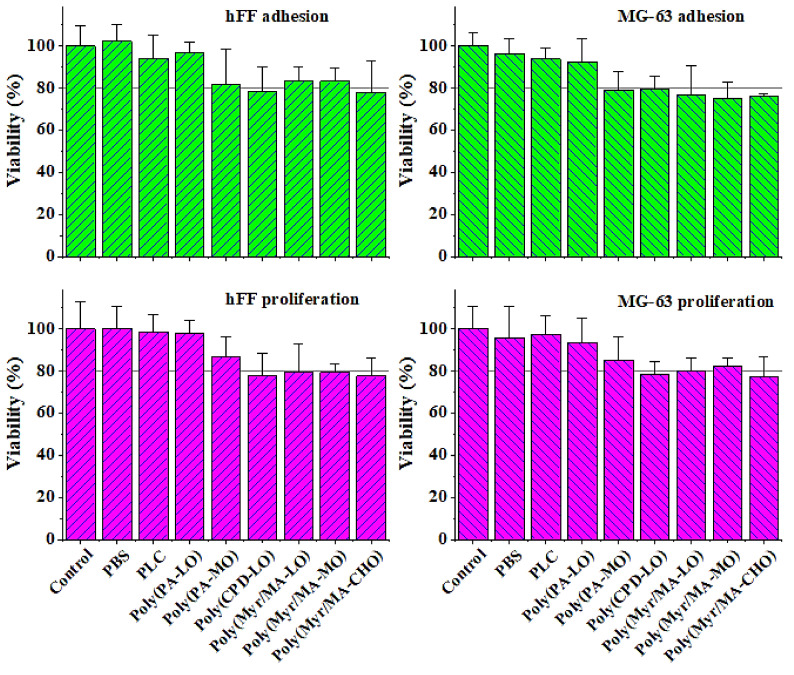
Cell adhesion and proliferation onto the surface of terpenoid-derived polymeric tablets. An MTT assay was performed at 24 h (adhesion test) and 96 h (proliferation test) of culture.

The cytotoxicity of the terpenoid-derived polymers was evaluated through an MTT assay. Fibroblast-type cells (hFF) and osteoblast-like cells (MG-63) were seeded on the surface of the polymeric tablet and were evaluated at 24 h and 96 h after exposure to determine the cytotoxic effect on cell adhesion and proliferation. Figure 10 show that PLC and poly(PA-LO) have no cytotoxic effect for the cell adhesion and proliferation of the fibroblasts hFF and MG-63. However, those polymers incorporating myrcene units showed a slight but non-significant decrease in cell adhesion and proliferation.

Terpenoids (i.e., terpenes and their oxygenated derivatives) are constituents of natural products (e.g., essential oils, flowers and medicinal plants) and have increasing applications in perfumery (aromas), the food industry (flavours), alternative medicine (aromatherapy) and the pharmaceutical industry (e.g., antibacterial and antineoplastic functions) [48]. Due to the use of terpenoids by humans, their neoplastic activity and genotoxicity have been evaluated in some cases. Thus, it has been demonstrated that limonene induces neoplasms in the kidneys of male rats in association with hyaline droplet nephropathy; and beta-myrcene produced kidney and liver cancers in rats and mice after in vivo exposure [49], but no mutagenic activity was found [50,51].

Recently, the cytotoxicity of beta-myrcene has been evaluated in-vitro using non-metabolizing cells such as the human peripheral blood mononuclear and metabolizing cells such as the human hepatoma cell line HepG2/C3A. Regarding the MTT assay, the results showed cytotoxic effects for leukocytes at 250 μg/mL and higher concentrations, while for HepG2/C3A cells, no cytotoxicity was observed relative to all tested concentrations (after 24 h exposure). However, under these experimental conditions, caution is recommended in the use of beta-myrcene since this compound produced genotoxic effects, especially after metabolic activation using human HepG2/C3A cells, which may be associated with carcinogenic and teratogenic effects previously reported in the literature [52].

The suitable polymerization and the low biodegradation rate of terpenoids can prevent their metabolization in the cells and, therefore, their cytotoxicity and potential genotoxic damage.

### 3.6. Blending of PBS with Poly(PA-LO)

**Figure 11:**
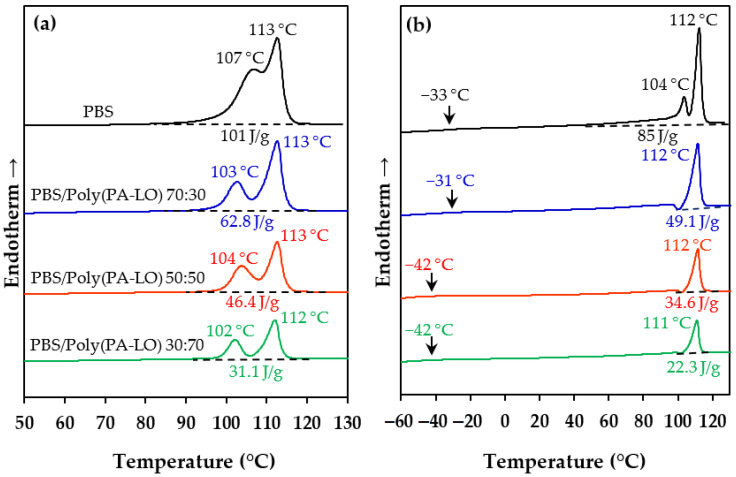
DSC first (**a**) and second (**b**) heating runs of PBS/poly(PA-LO) blends with the indicated weight ratios.

Poly(PA-LO) has been selected to be blended with PBS due to its higher glass transition temperature, presence of ester groups and good biocompatibility. Three PBS/poly(PA-LO) blends with different proportions (i.e., 30, 50 and 70 wt.% poly(PA-LO)) were compared with the neat PBS in terms of thermal properties (Figure 11).

The first heating run was characterized by two melting peaks related to PBS and no evidence of a polyterpene phase since its corresponding glass transition should be overlapped with the indicated melting peaks (Figure 11a). The DSC curves showed differences in the temperature of the former peak of the blends and the neat PBS (~103 °C vs. 107 °C), which is attributed to the formation of thinner lamellae in the blends. Note that the second and predominant peak (which is observed at a constant temperature around 113 °C for all samples) corresponds to the lamellar reorganization/recrystallization that occurs during the heating process. The second distinctive feature was a clear decrease in the global enthalpy of the blends by increasing the poly(PA-LO) content (global enthalpy decreased from 101 to 31.1 J/g when the poly(PA-LO) content increased from 0 to 70 wt.%). This feature is ascribed to a decrease in the global crystallinity of the blends as a consequence of the amorphous character of the polyterpene compound. In addition, a decrease in the melting enthalpy referred to the PBS content for the blends having 30 and 50 wt.% of poly(PA-LO) is resulted. In fact, correction of enthalpies considering the real PBS content gave values of 90, 93 and 104 J/g for the samples having 30, 50 and 70 wt.% of poly(PA-LO), respectively. Note that only in the third case the enthalpy was similar and even higher than that of the neat PBS (i.e., 101 J/g), as a consequence of a possible nucleation effect. For the other two compositions, the crystallization of PBS could slightly be hindered due to the presence of the second component.

Second heating runs (Figure 11b) pointed out the major difficulty in crystallizing from the melt with respect to solvent casting (i.e., enthalpies were always lower for the melt crystallized samples). The lamellar population with lower thickness was not thermally stable in the case of blends, and the neat polymer, thinner lamellae with a lower population were obtained via solvent casting. The crystallinity of PBS domains in the melt crystallized blends was always lower than that of the neat polyester. The corrected enthalpies of the blends were approximately 70 J/g (i.e., 70, 69 and 74 J/g for the blends with 30, 50 and 70 wt.% poly(PA-LO), respectively) as compared with 85 J/g for the neat PBS. Note that again a certain nucleation effect was found for the sample having a high poly(PA-LO) content, although in this case, the effect was less dramatic. Only the glass transition temperature of the PBS phase could be hardly observed around −31 to −42 °C. It should also be mentioned that no peak indicative of miscibility between the two polymeric components was detected.

**Figure 12:**
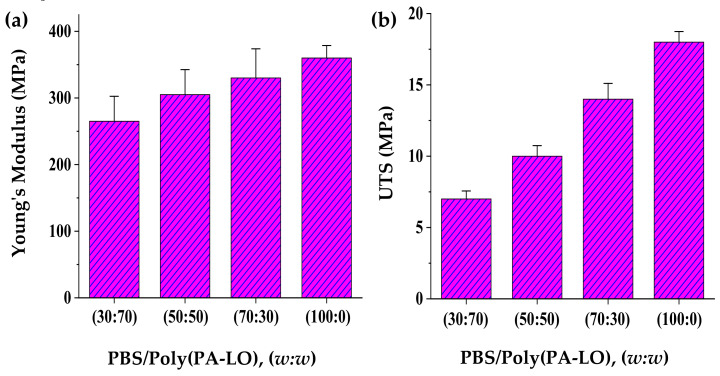
Comparison of the Young modulus (**a**) and the maximum strength (**b**) of neat PBS and the three studied PBS/poly(PA-LO) blends.

Mechanical properties of the PBS/poly(PA-LO) solvent casting films showed that the increase of the poly(PA-LO) content led to a decrease of the Young modulus (Figure 12a). Thus, values of 330, 305 and 265 MPa were determined for the blends with 30, 50 and 70 wt.% of poly(PA-LO), respectively. The decrease of the total crystallinity of the blends caused by a higher proportion of the amorphous polyterpene compound justifies the reduction of Young’s modulus. However, the modulus of the blend with 70 wt.% PBS was close to that of the neat polymer (360 MPa) since in this case, the stiffness of poly(PA-LO) could somewhat counterbalance the decrease of global crystallinity.

A similar trend was observed when ultimate strength (UTS) values were compared (Figure 12b). In this case, differences were more evident (i.e., values of 7, 10 and 14 MPa were obtained for the blends having 30, 50 and 70 wt.% of poly(PA-LO), while 18 MPa was measured for the neat polymer). The observed decrease was due to a reduction of the elongation at break caused by low compatibility between the two components.

All blends were hydrophobic since the contact angle of the neat PBS sample was 90° (solvent casting film), and after blending, a gradual increase of the angle with the copolymer content was observed. Thus, angles of 98°, 105° and 108° were found for the blends with 30, 50 and 70 wt.% poly(PA-LO), respectively.

Degradability was higher for the blends with a higher proportion of biodegradable PBS. Thus, for example, the weight loss detected after 21 days of exposure to the esterase medium at 37 °C was 5%, 8% and 12% for the blends having 30, 50 and 70 wt% of PBS, respectively.

**Figure 13:**
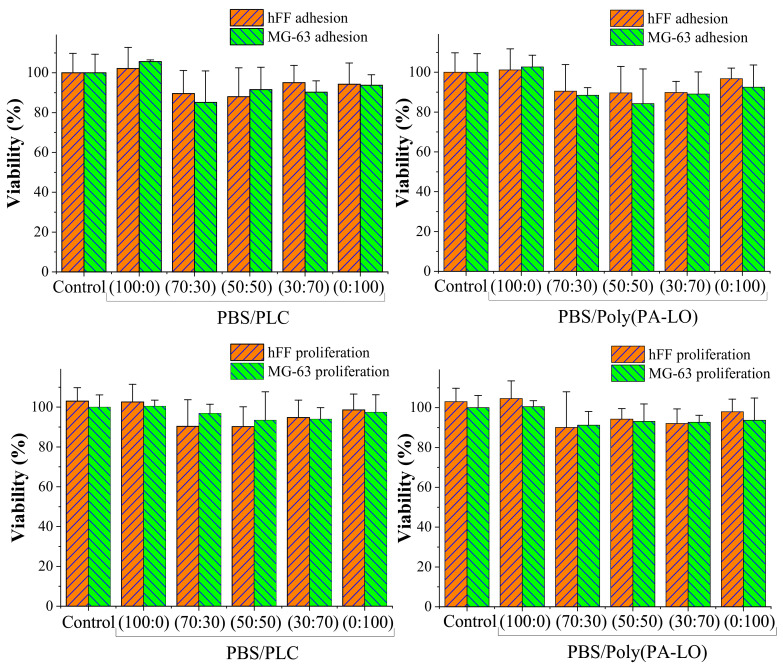
Cell adhesion and proliferation onto the surface of the PBS/PLC and PBS/poly(PA-LO) blend films. MTT assay was performed at 24 h (adhesion test) and 96 h (proliferation test) of culture.

Figure 13 show the results of the cell viability assay for cell adhesion and proliferation at the control endpoints. The fibroblasts hFF and MG-63 cells were cultured on the PBS/PLC and PBS/poly(PA-LO) blended films with different ratios of the polymers, prepared by solvent casting. In all cases, no cytotoxic effect was observed, demonstrating that the surfaces of these blends allow adequate cell adhesion and then these cells maintain their proliferative capacity on the surface without evidence of cytotoxicity.

It is also interesting to point out that results obtained for the different blends were almost similar to those found for pure polymers such as PBS, PLC and poly(PA-LO). Figure S5 show fluorescence images of proliferation cells on the different surfaces of the blend. In this case, the hFF fibroblasts adhered to the surface of the blends, making it possible to observe their well-extended fusiform shape.

Blends of PBS/PLC and PBS/poly(PA-LO) have been characterized by the restriction of the mobility of terpene oxide and aromatic units of phthalic, respectively. These restrictions in the mobility of the chains can favour the insolubility of the polymers and reduce their biodegradation. In this sense, this fact may favour biocompatibility because it is less likely that the terpene units were cleaved and solubilized. Therefore, the capacity of such units to enter the cellular metabolism and produce cytotoxic effects was dramatically decreased.

### 3.7. PBS/Poly(PA-LO) Blend Scaffolds from TIPS

The preparation of scaffolds constituted by the mixture of 70 wt.% of PBS and 30 wt.% of poly(PA-LO) was evaluated due to the interesting characteristic of the selected terpene derivative. In the present study, due to utilizing the 1,4 dioxane solvent with high crystallization temperature (i.e., 11.8 °C), a solid–liquid phase separation through crystallization of the solvent was expected during cooling. Nevertheless, in this system, there is a natural tendency for the semi-crystalline PBS polymer to be crystallized (i.e., solid-liquid phase separation through crystallization of the polymer) and also a tendency for the polymer solution to be separated into polymer-rich and polymer-lean phases during the cooling process (i.e., liquid-liquid phase separation). Hence, the studied system includes driving forces for three different mechanisms of phase separation. In this situation, each phase separation mechanism produces its own typical structure and affects the final morphology of the polymer matrix.

Crystallization of the solvent generally leads to large isotropic solid-walled pores with well-defined pore walls when a multidirectional cooling system is applied to the solution [53]. The pores formed through this mechanism had a geometry similar to the solvent crystallites. By applying a uniaxial cooling system, the solvent crystals grew along the cooling direction and produced an anisotropic microtubular structure in which the porosities formed by solvent crystals have been oriented towards the heat transfer direction [24,54]. The surface of such matrices typically has a ladder-like architecture in which repeating units of channels and partitioning walls are parallel and perpendicular to the temperature gradient [55]. Crystallization of the solvent rapidly advanced throughout the structure by expelling the polymer from the solvent crystallization front [54].

Liquid–liquid phase separation through the fast spinodal decomposition mechanism [56] produced a bicontinuous isotropic structure with the several-micron pores that may gradually coarsen to tens of microns if enough time was available [54,57]. Meanwhile, the polymer was able to crystallize slowly from the solution during or after the phase separation process in the polymer-rich phase, stabilizing the structure which has previously been formed by the phase separation. Crystallization or precipitation of the polymer from the solution may create different morphologies. By increasing the polymer concentration, these structures may vary from leafy structures characterized by randomly oriented and connected polymer leaves to spherulitic structures as an interconnected network of crystallites [58,59].

When the polymer solution involved driving forces for different phase separation mechanisms, the kinetics of phase separation specified whether the thermodynamically favored transition happens or not, and also to what extent the transition occurred [59]. Accordingly, by increasing the temperature gradient (i.e., faster cooling), the separation mechanisms which are kinetically fast can affect the structure (i.e., solvent crystallization and spinodal decomposition mechanisms). Therefore, the large pores formed through solvent crystallization and smaller porosities generated by the spinodal decomposition mechanism are expected to be observed at higher cooling rates.

**Figure 14:**
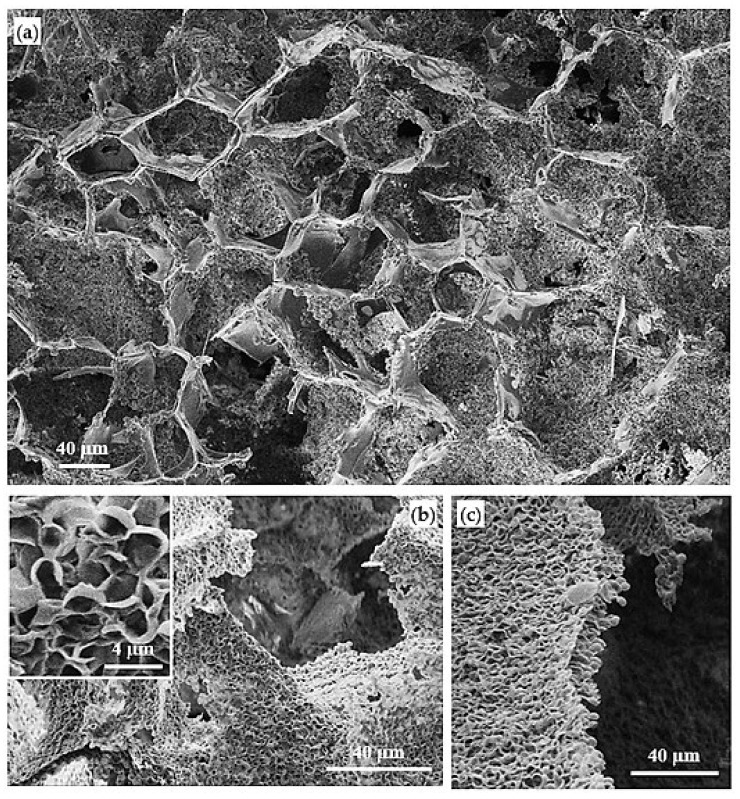
SEM micrograph of the PBS/poly(PA-LO) scaffold prepared using different cooling baths at: −74 °C (**a**), −20 °C (**b**,**c**). Inset shows the leafy structure of the corresponding scaffold at higher magnification.

As it is discernible in SEM micrographs (Figure 14), the scaffold prepared in the −74 °C bath includes large pores originating from crystallization of 1,4 dioxane. Moreover, the small pores formed by the spinodal decomposition mechanism that did not have enough time to grow are observed as several-micron orifices in the pore walls and play a role as interconnectivities (Figure 14a). The domination of large pores from solvent crystallization was decreased in the polymer matrix prepared at the slower cooling condition in the −20 °C bath (Figure 14b,c), while the spinodal-originated porosities were slightly coarsened. In this case, the texture of the pore walls was composed to a great extent of polymer leaves formed by crystallization of the polymer, a feature that is more detectable at higher magnification (inset of Figure 14b). Concerning their dimensions, these polymer leaves are supposed to be stacked lamellae that have developed with a significant curvature [58].

**Figure 15:**
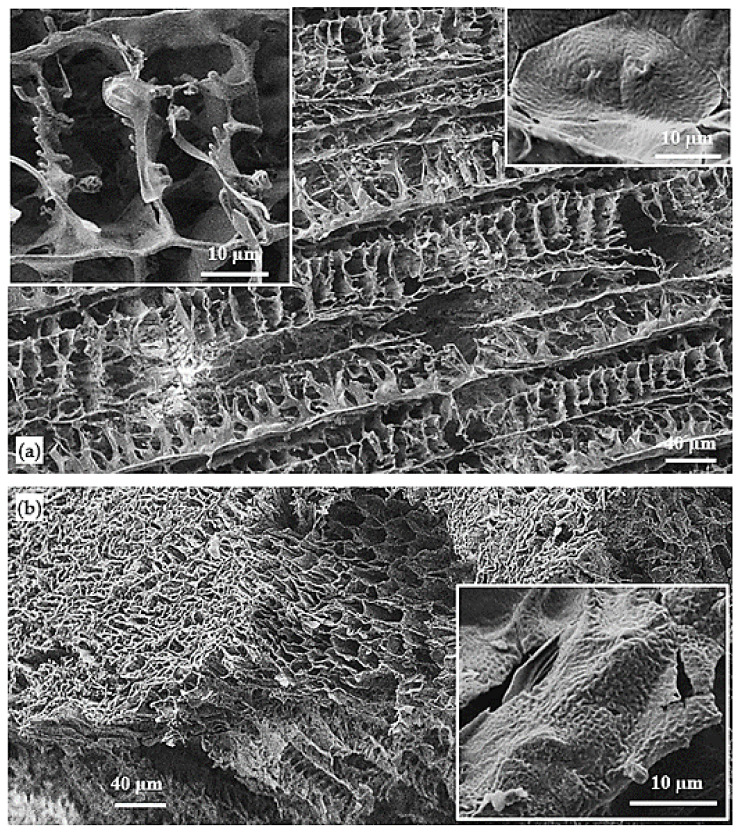
SEM micrograph of the PBS/poly(PA-LO) scaffold prepared under uniaxial temperature gradient condition at −196 °C: top surface (**a**), cross-section (**b**). Left inset of (**a**) shows a higher magnification of the corresponding surface. Right insets show spherulites with high magnification from the cross-section of oriented scaffolds made of neat PBS (**a**) [25] and PBS/poly(PA-LO) (**b**).

The surface image of the foam prepared using uniaxial temperature gradient in liquid nitrogen (Figure 15a, and the higher magnification image shown in the inset) exhibits the typical ladder-like structures with the repeating units and partitioning walls. The cross-section of this matrix demonstrates the microtubular architecture with the well-oriented porosities towards the heat transfer direction (Figure 15b).

It is interesting to note that the polymer has been crystallized in the form of polymer leaves at the lower temperature gradient but has formed a spherulitic structure using the uniaxial high temperature gradient. It can be speculated that the fast and orientational crystallization of 1,4 dioxane strongly avoids the polymer from the crystallization front and results in the formation of a more concentrated polymer-rich phase compared to that formed through liquid–liquid phase separation. The higher polymer concentration allows forming supermolecular spherulitic structures.

The spherulitic structure observed in the walls of the oriented pores of PBS/poly(PA-LO) scaffold (right inset of Figure 15b) is not as planar as the spherulites observed in the neat PBS matrix (right inset of Figure 15a) [24] and has a smaller size, slightly rougher texture and less integrity with the adjacent spherulites. These features indicate the imperfection of spherulite formation during the crystallization of the polymer in the polymer-rich separated phase. Furthermore, this result is in agreement with the previous calorimetric study of the studied blend that indicated a more difficult crystallization compared to that observed with the neat PBS.

Regarding the relatively low molecular weight of poly(PA-LO), it seems reasonable to assume that the small chains of this polymer can entangle between the PBS lamellae and cause structural disarrangement in the crystalline regions, limiting to some extent the ideal structures where the thickness of polymer leaves has been reduced (i.e., smaller lamella stacks) due to the presence of poly(PA-LO) in the polymer-rich phase. In addition, the presence of poly(PA-LO) has somewhat decreased the size and well-partitioning of large pores formed by solvent crystallization upon fast and multidirectional cooling.

## 4. Conclusions

Stiff biobased molecular chains can be obtained from the copolymerization of terpene oxides with either carbon dioxide or different dicarboxylic anhydrides having aromatic rings or aliphatic mono or bicycles. Copolymers offered the graduation of *T*_g_ from 75 °C to 127 °C. In addition, rings having a myrcene substitution showed a relatively low *T*_g_ as a consequence of the flexibility given by this lateral group.

The studied biobased polycarbonates and polyesters were thermally stable, and specifically, the type of terpene oxide was determined. Copolymers were completely amorphous when directly obtained from synthesis, although poly(limonene carbonate) rendered spherulites by the slow evaporation of diluted chloroform solutions. The capacity to crystallize this copolymer with all isopropene units having an R configuration was surprising and gave rise to a different structure than the orthorhombic packing previously reported for the racemic copolymer. The studied terpene-based copolymers were degraded with difficulty when typical hydrolytic and enzymatic media were considered. Nevertheless, GPC measurements demonstrated the progress of degradation in contrast with the low weight loss measurements, which reflect the low solubility of degraded fragments. Copolymers were fully biocompatible according to adhesion and proliferation assays performed with fibroblast cells. Only the presence of myrcene lateral groups caused certain toxicity.

Biobased terpene derivatives could be blended with poly(butylene succinate), giving rise to biocompatible and degradable materials. PBS/poly(PA-LO) blends with a maximum content of the terpene derivative of 30 wt.% showed similar mechanical properties to the neat polyester as a consequence of two opposite effects: the stiffness of the terpene compound and the decrease of the crystallinity of the sample.

Scaffolds with a random or an oriented pore distribution could successfully be prepared from 1,4-dioxane solutions by the temperature-induced phase separation method. The control of the cooling process of the initial homogenous solution was fundamental to vary the type of pore distribution, the size of pores and even the development of crystalline entities.

In summary, the studied biobased terpene derivatives showed a promising potential to be employed through polymer blending for biomedical applications. The high capacity to tune properties (e.g., glass transition temperature and hydrophobicity) and the well-proven ability to render porous scaffolds are significant. Furthermore, the rigid nature of the evaluated polyterpenes should be ideal for increasing stiffness and could lead to materials with similar characteristics to some human tissues (e.g., myocardium). In this sense, it seems necessary to take advantage of the high potential derived from the rigid molecular nature of the new polymers, explore the influence of the molecular weight in other to overcome some of the present limitations and finally, to evaluate the properties of blends with other biocompatible and biodegradable polymers such as PHAs and polylactide.

## Figures and Tables

**Table 1:** Solubility of the selected terpene derivatives in common organic solvents.

Solvent ^1^	Poly (PA-LO)	PLC	Poly (PA-MO)	Poly (Myr/MA-LO)	Poly (Myr/MA-MO)	Poly (CPD-LO)	Poly (Myr/MA-CHO)
CHCl_3_	++	++	++	++	++	++	++
TFA	++	++	++	++	++	++	++
HFIP	++	++	++	++	++	++	++
1,4 dioxane	++	++	++	++	++	++	++
THF	++	++	++	++	++	++	++
DMF	++	+	++	++	++	++	++
DMSO	+	+	-	++	-	+	--
Formic Acid	+	-	--	+	--	+	--
Formamide	-	-	-	-	-	-	-
Methanol	--	--	--	++	--	-	--
Ethanol	--	--	--	++	+	--	++

^1^ Code: ++: Soluble at room temperature; +: Soluble at higher temperatures or high-speed stirring/shaking; -: Insoluble; --: Very insoluble.

**Table 2:** GPC data of the selected terpene derivatives.

	Poly (PA-LO)	PLC	Poly (PA-MO)	Poly (Myr/MA-LO)	Poly (Myr/MA-MO)	Poly (CPD–LO)	Poly (Myr/MA-CHO)
*M_n_* (g/mol)	4000	3600	3900	4000	3900	3800	4100
*M_w_* (g/mol)	9200	7600	9000	9600	9700	8800	9100
PDI	2.3	2.1	2.3	2.4	2.5	2.3	2.2

## Supplementary Materials

The following are available online at https://www.mdpi.com/article/10.3390/polym14010161/s1, Figure S1: GPC curves for selected samples, Figure S2: Solvent casting films obtained from selected PBS blends and representative pressed tablet of pure poly(PA-LO), Figure S3: Contact angles determined from the different pressed tablets of the studied terpene derivatives, Figure S4: X-ray diffraction profiles of the as-synthesized powdered samples of terpene derivatives, Figure S5: Fluorescence images of the proliferated hFF fibroblasts on the different surfaces of representative PBS blends.
