# Supplementary material for: Biobased Terpene Derivatives: Stiff and Biocompatible Compounds to Tune Biodegradability and Properties of Poly(butylene succinate)
Source: Polymers (Basel). 2021 Dec 31;14(1):161. doi: 10.3390/polym14010161 (PMC8747377; doi:10.3390/polym14010161)
Supplement: Supplementary file 1 [file polymers-14-00161-s001.zip › polymers-1525430-supplementary.pdf]

Supplementary Materials:

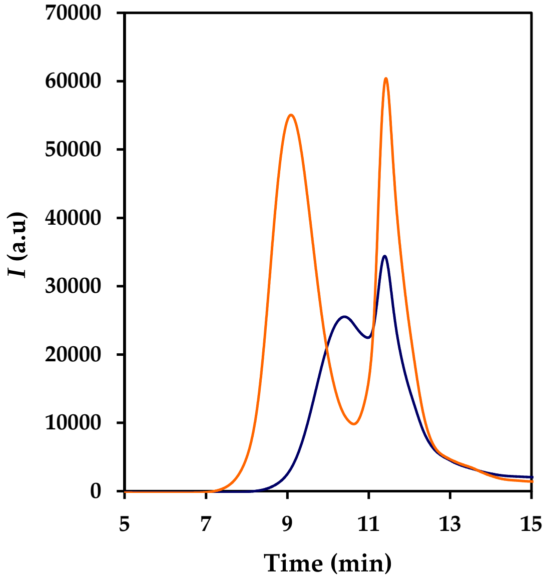

Figure S1. GPC curves for poly(Myr/MA-LO) (orange) and PLC (blue) samples. Peaks at the high retention time (i.e., 12 min) corresponds to the salts of the elution medium.

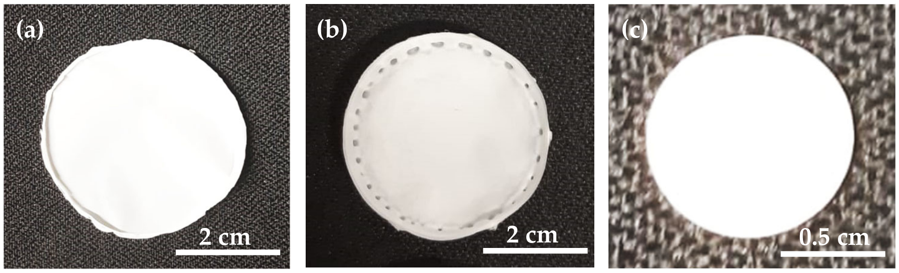

Figure S2. Solvent casting films obtained from PBS/poly(PA-LO) 70:30 (**a**), PBS/poly(PA-LO) 50:50 (**b**) and a pressed tablet of pure poly(PA-LO) (**c**).

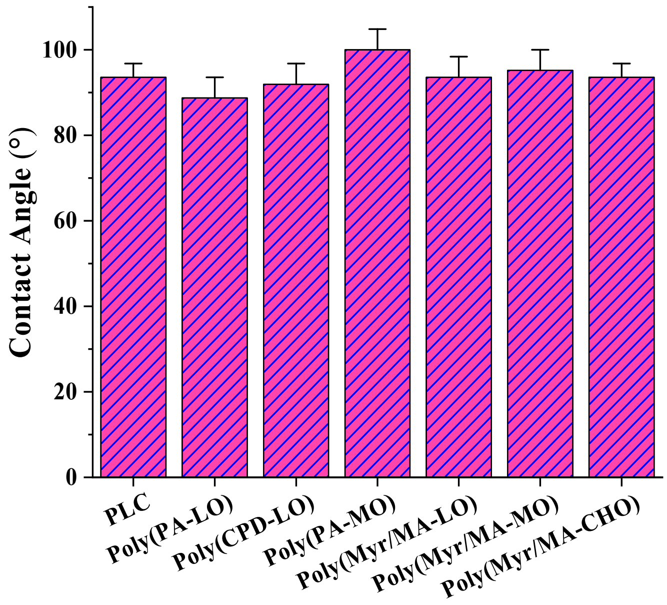

Figure S3. Contact angles determined from the different pressed tablets of the studied terpene derivatives.

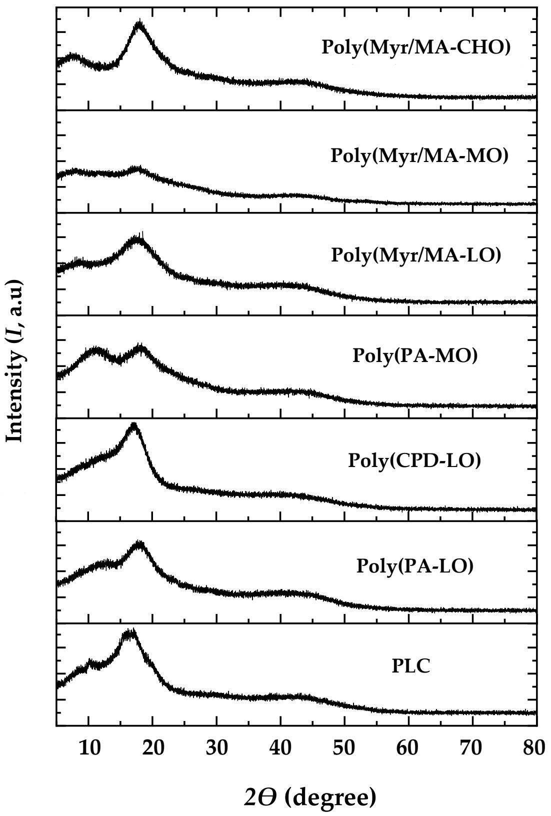

Figure S4. X-ray diffraction profiles of the as synthesized powdered samples of the different terpene derivatives.

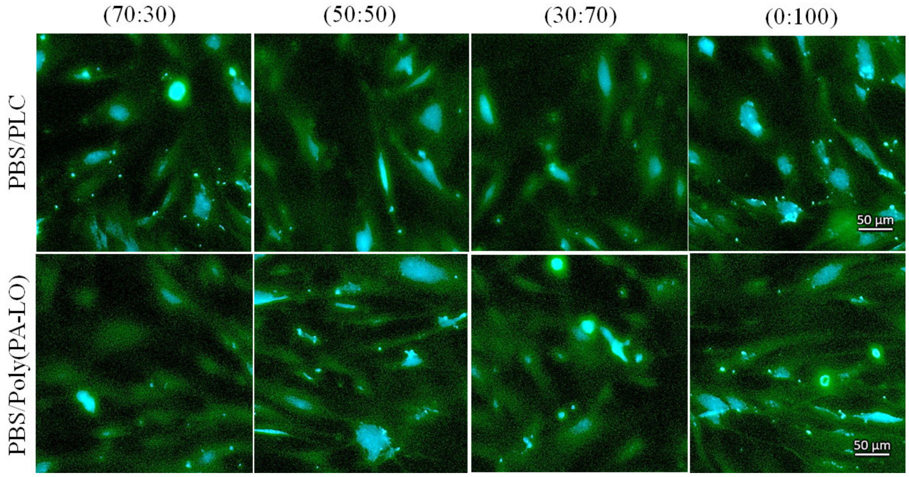

Figure S5. Fluorescence images of the proliferated hFF fibroblasts on the different surfaces of the PBS/PLC and PBS/poly(PA-LO) blends. The cytoplasm was stained with phalloidin (green) and the nucleus with Hoechst (blue).
